# Awareness and Prescribing Patterns of Acute and Preventive Migraine Medications Among General Practitioners: The Kawasaki Medical Association Study

**DOI:** 10.1155/prm/4962211

**Published:** 2026-10-07

**Authors:** Hisanao Akiyama, Haruka Ono, Yukari Komine, Yoko Nakano, Yoshihisa Yamano

**Affiliations:** ^1^ Department of Neurology St. Marianna University School of Medicine Kawasaki Kanagawa Japan marianna-u.ac.jp

**Keywords:** acute and preventive migraine medications, anti-CGRP monoclonal antibodies, awareness and prescribing patterns, general practitioners, triptans

## Abstract

**Aim:**

In Japan, the introduction of anti‐calcitonin gene‐related peptide (CGRP) monoclonal antibodies and the 5‐HT1F receptor agonist lasmiditan has transformed migraine treatment. However, medical consultation and prescription rates for migraine remain low. This study aimed to clarify the awareness and prescribing patterns of acute and preventive migraine medications among physicians in Kawasaki, Japan.

**Methods:**

A questionnaire survey of 1274 physicians registered with the Kawasaki Medical Association was conducted from December 2024 to February 2025. Responses were collected via fax or web form, and data were analyzed according to medical specialty.

**Results:**

A total of 288 physicians responded (response rate, 22.6%). General internists accounted for 49.3%, followed by neurologists (5.9%) and neurosurgeons (3.8%). For acute treatment, 65.9% of the respondents primarily prescribed acetaminophen or nonsteroidal anti‐inflammatory drugs. Although 87.8% were aware of triptans, only 64.1% prescribed them. Awareness and prescription of lasmiditan and anti‐CGRP monoclonal antibodies were particularly low among nonspecialists and were significantly lower than those among specialists (*p* < 0.008). In the multivariable analysis, specialty was not independently associated with triptan prescribing, whereas specialists were markedly more likely to prescribe anti‐CGRP monoclonal antibodies (adjusted OR 42.6).

**Conclusion:**

In Kawasaki, awareness and use of new migraine therapies remain limited, especially among nonspecialists. Specialty independently influenced the prescription of anti‐CGRP monoclonal antibodies but not triptans, highlighting the need for enhanced education and collaboration with headache specialists to improve migraine care.

**Clinical Implications**
• Limited awareness and prescribing patterns of migraine‐specific drugs among general practitioners restrict optimal patient care.
• Education on triptans, lasmiditan, and anti‐CGRP monoclonal antibodies is essential.
• Improving referral pathways to headache specialists may expand access to advanced therapies.
• Community‐based educational initiatives could enhance treatment quality and reduce migraine‐related disability.

## 1. Introduction

In the treatment of migraine, a common disease worldwide, a paradigm shift has occurred following large‐scale clinical trials [1–8], with anti‐calcitonin gene‐related peptide (CGRP) monoclonal antibodies being launched in Japan as preventive therapy in 2021 and the 5‐HT1F receptor agonist lasmiditan as an acute treatment in 2022. However, despite subsequent longstanding efforts by the Japanese Headache Society, healthcare professionals, and industry to encourage patients with headache disorders to seek medical attention and attend regular follow‐up visits, low rates of medical consultation among headache sufferers and the underprescription of migraine medications, both acute and preventive, continue to be reported [9–11]. Although insufficient patient engagement may partly explain this situation, it is also possible that issues on the healthcare provider side contribute, including a lack of familiarity with migraine‐specific medications among general practitioners, who are presumed to be responsible for managing most headache patients.

Against this background, we conducted a questionnaire survey among general practitioners belonging to the Kawasaki Medical Association to assess their awareness and prescribing practices regarding migraine medications. Our aim was to identify potential healthcare system‐related factors contributing to the persistently low rates of regular medical follow‐up among patients with headache disorders.

## 2. Methods

Between December 2024 and February 2025, a 3‐month questionnaire survey was administered to 1274 physicians of the Kawasaki Medical Association who had registered their e‐mail addresses on the association’s website. A mass e‐mail was sent to these physicians requesting participation in a survey regarding their awareness and prescribing practices of acute and preventive migraine medications. Responses were obtained via fax or a web‐based form from physicians who consented to participate. Based on these responses, we examined the awareness and prescription patterns of these migraine medications across physician groups, including neurologists and neurosurgeons, who are presumed to include a higher proportion of headache specialists, and general internists or physicians from other specialties, who are presumed to include fewer headache specialists. This study is reported in accordance with the STROBE guidelines for cross‐sectional observational studies.

### 2.1. Kawasaki

Kawasaki is a city in the Kanto region of Japan, situated between Tokyo and Yokohama. Adjacent to the central area of the Tokyo metropolitan region, the largest urban area in Japan, Kawasaki has a particularly high population density. As of 2025, the population is approximately 1.55 million, distributed across seven administrative wards. Similar to the national trend, the birth rate is low. The proportion of older adults (≥ 65 years) is approximately 20%, slightly below the national average, while the number of younger residents is relatively high, reflecting characteristics typical of an urban area.

### 2.2. Kawasaki Medical Association

The Kawasaki Medical Association was established in 1924, coinciding with the enactment of Kawasaki’s municipal system, and was reorganized as the Incorporated Association Kawasaki Medical Association in 1947. It transitioned to a Public Interest Incorporated Association in 2013. It currently consists of more than 1400 member physicians from over 800 medical institutions across the seven districts of Kawasaki City, representing a wide range of medical specialties.

### 2.3. Questionnaire Items

The seven question items are shown in Table 1.

**TABLE 1:** The seven question items used in the physician survey.

	
Q1	Age, sex, and medical specialty

Q2	For the acute (attack phase) treatment of migraine, do you mainly prescribe acetaminophen or nonsteroidal anti‐inflammatory drugs (NSAIDs)?
	Yes/no/other (free description)

Q3	Are you aware of triptans, which have been marketed as acute migraine medications?
	Yes/no/other (free description)
	Have you actually prescribed triptans?
	Yes/no/other (free description)

Q4	Are you aware of lasmiditan, a newly marketed acute migraine medication?
	Yes/no/other (free description)
	Have you actually prescribed lasmiditan?
	Yes/no/other (free description)

Q5	Are you aware of conventional preventive migraine medications included lomerizine, valproic acid, propranolol, and amitriptyline?
	Yes/no/other (free description)
	Have you actually prescribed these preventive medications?
	Yes/no/other (free description)

Q6	Are you aware of the newly marketed anti‐CGRP monoclonal antibody subcutaneous formulations for migraine prevention?
	Yes/no/other (free description)
	Have you actually prescribed these anti‐CGRP monoclonal antibodies?
	Yes/no/other (free description)

Q7	Are you aware that anti‐CGRP monoclonal antibody subcutaneous formulations have high efficacy and few adverse effects?
	Yes/no/other (free description)

*Note:* Each item includes a yes/no response option with an additional free‐description field when applicable.

Conventional preventive medications included lomerizine, valproic acid, propranolol, and amitriptyline, and these agents were analyzed as a single category in the statistical evaluation.

### 2.4. Statistical Analysis

Fisher’s exact test was used for comparisons among medical specialties because the expected frequencies were less than 5, with some cells containing 0 cases, for (i) prescription of acetaminophen and nonsteroidal anti‐inflammatory drugs (NSAIDs), (ii) awareness and prescription of acute‐phase medications such as triptans and lasmiditan, (iii) awareness and prescription of conventional preventive medications and anti‐CGRP monoclonal antibodies, (iv) referrals for prescribing anti‐CGRP monoclonal antibodies, and (v) awareness of the benefits of anti‐CGRP monoclonal antibodies. Pearson’s chi‐square test was also applied for overall comparisons across the four specialty groups when expected cell counts permitted. For pairwise comparisons between specialties (six combinations), Fisher’s exact test was used and Bonferroni correction was applied to adjust for multiple testing (corrected significance threshold *α* = 0.0083).

To evaluate the broader trends in awareness, prescribing behavior, and referral patterns, specialties were further grouped into nonspecialists (general internists and other specialties; GI + O) and specialists (neurologists and neurosurgeons; N + NS), and 2 × 2 comparisons were performed using the chi‐square or Fisher’s exact test as appropriate. Multivariable logistic regression analyses were additionally performed to assess whether specialty independently predicted prescribing behavior for triptans or anti‐CGRP monoclonal antibodies, as well as referral behavior to specialists, after adjusting for age and sex. All statistical analyses were performed using IBM SPSS Statistics for Windows ver. 26 (IBM Inc., Armonk, NY). All statistical tests were two‐sided, and *p* values < 0.05 were considered statistically significant. Because some questionnaire items had missing responses, denominators vary across analyses.

### 2.5. Ethics Statement

This study was approved by the Bioethics Committee of St. Marianna University School of Medicine (approval no. 6682). Informed consent was obtained from all participants included in this study.

## 3. Results

### 3.1. Background Characteristics of the Responding Physicians

Of the 1274 physicians invited to participate in the survey, 288 physicians responded (22.6%). Among the respondents, 225 were men (78.1%), 57 were women (19.8%), and 6 did not report their gender. The respondents’ ages ranged from 33 to 89 years, and the mean age was 58.8 ± 10.7 years. Regarding medical specialty, only 17 physicians (5.9%) were neurologists and 11 (3.8%) were neurosurgeons, both of which were considered to include a higher proportion of headache specialists. In contrast, 142 physicians (49.3%) were general internists and 118 (41.0%) belonged to other specialties, which were considered to include fewer headache specialists. Among these “other specialties,” the most common were pediatrics (*n* = 23), obstetrics and gynecology (*n* = 20), otolaryngology (*n* = 14), ophthalmology (*n* = 12), orthopedics (*n* = 8), and psychiatry (*n* = 7) (Table 2).

**TABLE 2:** Background characteristics of the responding physicians.

Other specialties	Number
Pediatrics	23
Obstetrics and gynecology	20
Otolaryngology	14
Ophthalmology	12
Orthopedics	8
Psychiatry	7
Urology	5
Cardiology	4
Gastroenterology	3
General surgery	3
Dermatology	2
Plastic surgery	2
Rehabilitation	2
Gastrointestinal surgery	2
Health checkup/occupational health	2
Thoracic surgery	1
Anesthesiology	1
Family medicine	1
Radiology	1
Breast surgery	1
Other	1
Blank	3

*Note:* Values are presented as number of physicians by specialty.

### 3.2. Awareness and prescribing patterns of Acute Migraine Medications

Overall, 189 physicians (65.9% of 287 respondents to this item) reported that they mainly prescribed acetaminophen or NSAIDs for acute migraine treatment. By specialty, the proportion was 67.6% among general internists and 71.2% among other specialties, whereas it was lower among neurosurgeons (54.5%) and particularly low among neurologists (17.6%). A significant association was observed between the prescription of acetaminophen or NSAIDs and the medical specialty (*p* = 2.1 × 10^−4^) (Figure 1). Pairwise comparisons using Fisher’s exact test revealed that neurologists had significantly lower prescribing rates compared with general internists (*p* = 0.000003) and other specialties (*p* = 0.0000002). Neurosurgeons also tended to prescribe these agents less frequently compared with general internists (*p* = 0.049) and other specialties (*p* = 0.03), although the differences did not remain significant after Bonferroni correction (*α* = 0.0083). No significant differences were observed between general internists and other specialties (*p* = 0.47) or between neurologists and neurosurgeons (*p* = 0.13). To further evaluate the overall trend, the four specialties were grouped into two categories: general internists plus other specialties (GI + O) and neurologists plus neurosurgeons (N + NS). The proportion of physicians who prescribed acetaminophen or NSAIDs was significantly higher in the GI + O group (69.2%, 180/260) than in the N + NS group (32.1%, 9/28) (*χ* ^2^ = 20.7, *p* < 0.00001; Fisher’s exact test *p* < 0.00001).

**FIGURE 1:**
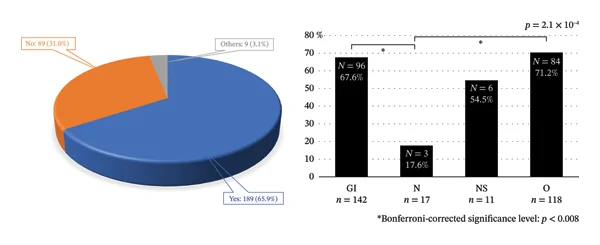
Proportion of physicians prescribing acetaminophen or NSAIDs for acute migraine by specialty (*n* = 287). Overall, 65.9% of physicians reported using these agents as first‐line therapy. General internists (GI) and other specialties (O) showed higher prescribing rates, whereas neurosurgeons (NS) and neurologists (N) demonstrated lower rates. A significant association was observed between medical specialty and prescribing behavior (*p* = 2.1 × 10^−4^). Pairwise comparisons were evaluated using Fisher’s exact test with Bonferroni correction (significant at *p* < 0.008). ∗Bonferroni‐corrected significance level: *p* < 0.008. NSAID, nonsteroidal anti‐inflammatory drug.

Regarding triptans, 253 physicians (87.8%) were aware of these agents. However, only 184 physicians (64.1%) reported prescribing them, indicating a clear discrepancy between awareness and actual prescribing. Furthermore, awareness of lasmiditan, a newly marketed acute migraine medication, was low at 105 physicians (36.6%), and the number of physicians who had prescribed it was even smaller at 41 (14.2%) (Figure 2).

**FIGURE 2:**
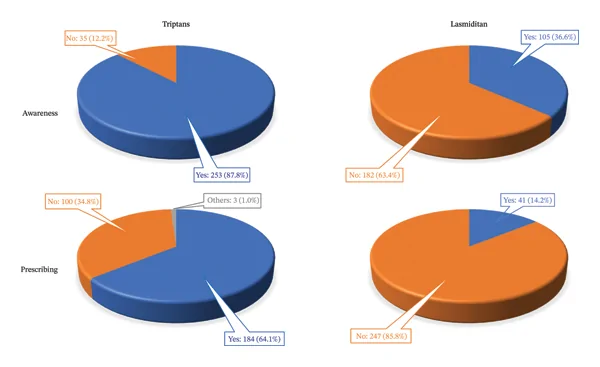
Awareness and prescription of triptans (*n* = 288, 287) and lasmiditan (*n* = 287, 288). Although 87.8% of respondents were aware of triptans, only 64.1% prescribed them, demonstrating a clear gap between awareness and actual prescribing behavior. Awareness of lasmiditan was low (36.6%), and its prescription was even lower (14.2%), indicating limited uptake of this newly marketed acute migraine medication.

For triptans, although 78.0% of physicians in other specialties were aware of them, only 36.4% prescribed them. For lasmiditan, both awareness (40.8% in general internal medicine; 17.8% in other specialties) and prescription (11.3% and 2.5%, respectively) were particularly low. Significant associations were observed between awareness, prescription, and department (*p* = 4.4 × 10^−4^, *p* = 1.2 × 10^−15^, *p* = 1.9 × 10^−13^, *p* < 2 × 10^−16^). Pairwise comparisons showed that neurologists and neurosurgeons had significantly higher awareness and prescribing rates of both triptans and lasmiditan compared with general internists and other specialties (all *p* < 0.01 after Fisher’s exact test). No significant differences were observed between neurologists and neurosurgeons or between general internists and other specialties. When specialties were grouped into the abovementioned two categories (GI + O vs. N + NS), the N + NS group demonstrated markedly higher awareness and prescribing rates for both triptans and lasmiditan (all *p* < 0.00001) (Figure 3).

**FIGURE 3:**
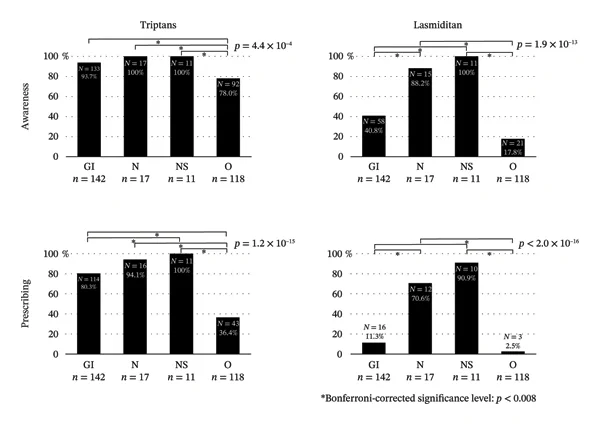
Awareness and prescription of triptans and lasmiditan by specialty (*n* = 288). Awareness of triptans was relatively high across all specialties, although prescribing remained low among nonspecialist groups. For lasmiditan, both awareness and prescribing were markedly lower among general internists and other specialties, whereas neurologists and neurosurgeons showed substantially higher levels. A significant association was observed between specialty and both awareness and prescribing (*p* values shown in the figure). Pairwise comparisons were evaluated using Fisher’s exact test with Bonferroni correction (significant at *p* < 0.008). GI, general internists; N, neurologists; NS, neurosurgeons; O, others.

### 3.3. Awareness and prescribing patterns of Preventive Migraine Medications

A total of 218 physicians (76%) were aware of conventional oral preventive medications for migraine, such as lomerizine and valproic acid. In this study, conventional preventive medications included lomerizine, valproic acid, propranolol, and amitriptyline, and these agents were treated as a single category in the analysis. However, only 115 physicians (40.1%) reported prescribing them. Awareness of anti‐CGRP monoclonal antibodies was even lower at 135 physicians (47%), and only 30 physicians (10.5%) had prescribed them (Figure 4).

**FIGURE 4:**
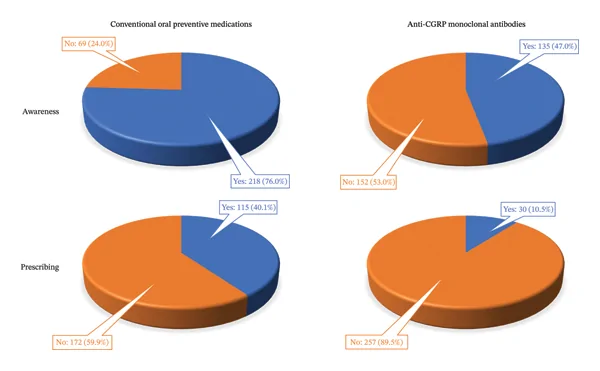
Awareness and prescription of conventional oral preventive medications and anti‐CGRP monoclonal antibodies (*n* = 287). While 76.0% of physicians were aware of agents such as lomerizine and valproic acid, only 40.1% prescribed them, showing a substantial gap between awareness and use. Awareness was limited (47.0%), and prescription was very low (10.5%), reflecting restricted adoption of these newer preventive therapies. CGRP, calcitonin gene‐related peptide.

Specialty‐specific analysis of preventive medications showed that awareness of conventional oral preventive agents was particularly low among other specialties (61.9%), as well as their prescription (16.9%). Even among general internists, the prescription rate (47.9%) was approximately half the awareness rate (82.4%). For anti‐CGRP monoclonal antibodies, both awareness and prescription were particularly low among general internists and other specialties. Even neurologists, although 94.1% were aware of these agents, only 52.9% reported prescribing them. Significant associations were observed among awareness, prescription, and department (*p* = 9.0 × 10^−6^, *p* = 3.6 × 10^−16^, *p* = 3.8 × 10^−10^, *p* < 2 × 10^−16^) (Figure 5). Pairwise comparisons showed that neurologists and neurosurgeons had significantly higher awareness and prescribing rates of both conventional preventive agents and anti‐CGRP monoclonal antibodies compared with general internists and other specialties (all Bonferroni‐corrected *p* < 0.0083). No significant differences were observed between neurologists and neurosurgeons or between general internists and other specialties. When specialties were grouped into two categories (GI + O vs. N + NS), the N + NS group demonstrated markedly higher awareness and prescribing rates for both preventive medication classes (all *p* < 0.00001).

**FIGURE 5:**
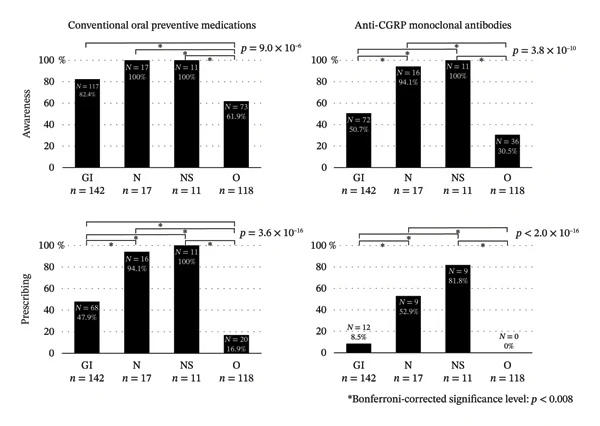
Awareness and prescription of preventive migraine medications by specialty (*n* = 288). Awareness and prescription of conventional oral preventive agents were lowest among other specialties, and prescription rates were substantially lower than awareness among general internists. For anti‐CGRP monoclonal antibodies, both awareness and prescribing were low across all nonneurologist groups, whereas neurologists and neurosurgeons showed the highest levels. A significant association was observed between specialty and awareness and between specialty and prescribing (*p* values shown in the figure). Pairwise comparisons were evaluated using Fisher’s exact test with Bonferroni correction (significant at *p* < 0.008). CGRP, calcitonin gene‐related peptide; GI, general internists; N, neurologists; NS, neurosurgeons; O, other.

### 3.4. Referral Rates to Headache Specialists

Because anti‐CGRP monoclonal antibodies require specialist involvement for prescription, we examined referral rates to headache specialists. Referral rates were low—33.8% among general internists and 41.5% among other specialties—indicating that referrals for the purpose of initiating anti‐CGRP monoclonal antibody therapy were significantly uncommon (*p* = 9.1 × 10^−5^). Pairwise comparisons showed that referral rates were significantly lower among neurologists and neurosurgeons than among general internists and other specialties (all Bonferroni‐corrected *p* < 0.0083). No significant differences were observed between neurologists and neurosurgeons or between general internists and other specialties. When specialties were grouped into two categories (GI + O vs. N + NS), referral rates were markedly higher in the GI + O group (97/260) than in the N + NS group (0/28) (*p* < 10^−12^), indicating that nonspecialists rarely referred patients despite being the group expected to do so.

### 3.5. Awareness of the High Efficacy and Low Incidence of Adverse Effects of Anti‐CGRP Monoclonal Antibodies

We also assessed whether physicians were aware that anti‐CGRP monoclonal antibodies have high efficacy and few adverse effects (Figure 6). Only 102 physicians (35.5%) were aware of these characteristics, with awareness being low among general internists (37.3%) and other specialties (19.5%) in particular. A significant association was observed between awareness of the benefits of anti‐CGRP monoclonal antibodies and department (*p* = 2.4 × 10^−12^). Pairwise comparisons showed that neurologists and neurosurgeons had significantly higher awareness compared with general internists and other specialties (all Bonferroni‐corrected *p* < 0.0083). No significant differences were observed between neurologists and neurosurgeons or between general internists and other specialties. When specialties were grouped into two categories (GI + O vs. N + NS), the N + NS group demonstrated markedly higher awareness of the benefits of anti‐CGRP monoclonal antibodies (*p* < 0.00001).

**FIGURE 6:**
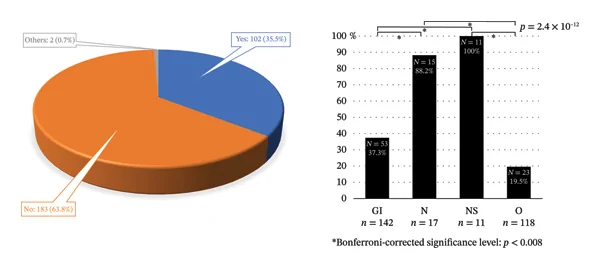
Awareness of high efficacy and low adverse effect of anti‐CGRP monoclonal antibodies (*n* = 287). Only 35.5% of physicians were aware of these characteristics, with particularly low awareness among general internists and other specialties. Neurologists and neurosurgeons showed the highest awareness levels. Pairwise comparisons were evaluated using Fisher’s exact test with Bonferroni correction (significant at *p* < 0.008). CGRP, calcitonin gene‐related peptide; GI, general internists; N, neurologists; NS, neurosurgeons; O, other.

### 3.6. Multivariable Analysis for Prescribing Behavior for Triptans or Anti‐CGRP Monoclonal Antibodies, and Referral Behavior to Specialists

Multivariable logistic regression analyses were conducted to determine whether specialty independently contributed to prescribing behavior for triptans and anti‐CGRP monoclonal antibodies, as well as referral behavior to specialists. Although no significant difference was observed between specialists and nonspecialists in triptan prescribing, specialists were markedly more likely to prescribe anti‐CGRP monoclonal antibodies (adjusted OR 42.60, 95% CI 14.48–125.30, *p* < 0.001). Age showed a slight negative association with CGRP monoclonal antibody prescribing (adjusted OR 0.94, 95% CI 0.89–0.99, *p* = 0.015), whereas sex was not significantly associated with prescribing behavior. Referral behavior to specialists also demonstrated only a minimal negative association with age (adjusted OR 0.98, 95% CI 0.95–0.99, *p* = 0.039). Although descriptive analyses showed higher triptan prescribing rates among specialists, this association did not remain significant after adjusting for age and sex, suggesting that the apparent difference in triptan prescribing may be partly explained by confounding factors such as age distribution across specialties.

## 4. Discussion

This study provides a detailed characterization of migraine management in community healthcare by examining the awareness and prescribing patterns of acute and preventive migraine medications among general practitioners belonging to the Kawasaki Medical Association. The findings suggest that the issue extends beyond simple gaps in drug awareness and reflects a complex interplay of structural challenges in Japan’s community healthcare system.

General practitioners play a central role in the early management of episodic and chronic migraine. Insufficient engagement at the primary‐care level may delay the initiation of appropriate acute and preventive therapy, increasing the risk of chronification and medication overuse. Although the fundamental clinical hallmarks of migraine have been clearly described in recent literature [11], they remain underrecognized in primary‐care settings, and international studies consistently show that primary‐care physicians lack confidence in headache diagnosis and treatment [12–15]. This shared pattern across countries underscores the need for structured, evidence‐based educational programs tailored for general practitioners. These concerns align with global reports indicating that primary‐care physicians often underestimate migraine severity and may not apply evidence‐based diagnostic or therapeutic algorithms.

Although awareness of triptans was high, their prescription rate remained insufficient in this study. This discrepancy between awareness and actual prescription aligns with the well‐documented pattern of undertreatment of migraine both in Japan and internationally. In the United States, despite triptans being introduced nearly 30 years ago, many patients still lack access to appropriate acute treatment [16–18]. Similar trends have been reported in Europe, where NSAIDs and acetaminophen are frequently used as first‐line agents in primary‐care settings [18]. In Japan, the OVERCOME Japan studies clearly demonstrated low consultation rates and low triptan use among migraine patients [10], consistent with our findings. Contributing factors may include excessive concern regarding triptan contraindications, and in middle‐aged and older patients, higher cardiovascular risk may make physicians more hesitant to prescribe triptans, as well as a tendency among physicians to underestimate migraine because it is not life‐threatening. Furthermore, Japan’s diagnostic culture—where neuroimaging is often emphasized and normal imaging findings may lead to reassurance without therapeutic intervention—can reduce opportunities for appropriate treatment and contribute to underutilization of migraine‐specific medications. Although specialists showed higher triptan prescribing rates in descriptive analyses, this association did not remain significant in the multivariable model, indicating that specialty itself was not an independent predictor once age and sex were accounted for.

The low awareness and prescription rates of newer agents such as lasmiditan and anti‐CGRP monoclonal antibodies indicate an even more serious issue. In particular, anti‐CGRP monoclonal antibodies have driven a paradigm shift in preventive migraine therapy worldwide and have demonstrated high efficacy in patients who respond inadequately to conventional treatments [1–6]. However, in the present study, awareness among nonheadache specialists was extremely low, and actual prescribing was nearly nonexistent. This suggests not only a lack of information but also the influence of Japan’s specialist‐centered healthcare structure. In Japan, anti‐CGRP monoclonal antibodies are prescribed in facilities where physicians meet specific qualification requirements defined in the optimal‐use guideline, which effectively limits their use to specialist‐centered settings.

Recent Japanese consensus statements position anti‐CGRP monoclonal antibodies as an important preventive treatment option for migraine [19], making the extremely low awareness and prescription among nonspecialists a critical gap in community migraine care [14, 19]. A recent cross‐sectional study demonstrated that tertiary centers were more likely to initiate CGRP‐directed therapies, whereas primary‐care physicians relied more heavily on conventional preventives, paralleling our findings [14]. Regarding lasmiditan, although it represents a nonvasoconstrictive acute treatment option, its clinical relevance differs between countries: although lasmiditan has been discontinued in the United States, limiting its international availability [20], it remains clinically relevant in Japan, where real‐world data continue to support its effectiveness and safety [21]. Accordingly, continued educational efforts to improve awareness and prescription rates among nonheadache specialists are considered necessary. Nevertheless, given the uncertain outlook—such as the absence of large‐scale additional trials or new indication‐expansion efforts—the educational priority of lasmiditan may reasonably be positioned lower than that of triptans and anti‐CGRP monoclonal antibodies, which have stronger evidence bases and broader international relevance; however, its future clinical positioning in Japan remains unclear.

In the present study, only 35.4% of physicians understood the high efficacy and favorable safety profile of anti‐CGRP monoclonal antibodies, indicating insufficient dissemination of fundamental knowledge about these agents. Recent systematic reviews have demonstrated that anti‐CGRP monoclonal antibodies and gepants show favorable cardiovascular and cerebrovascular safety profiles even in patients with preexisting vascular risk factors [22–25]. These findings are consistent with evidence showing robust efficacy and safety across episodic and chronic migraine populations, including those with medication‐overuse headache [25]. Limited access to specialist‐centered educational opportunities likely contributes to this knowledge gap among general practitioners.

The low referral rate to specialists is another important concern [26]. Although specialist involvement is required for prescribing anti‐CGRP monoclonal antibodies, referral rates were only 33.8% among general internists and 41.5% among other specialties. These findings indicate insufficient recognition of the need for referral, a shortage of headache specialists in the community, or procedural barriers that complicate referrals. In the United States, only 4%–5% of migraine patients are seen by specialists [14], and similar structural issues may exist in Japan. The OVERCOME (Japan) second study further demonstrated that many Japanese migraine patients first consult general practitioners but often do not receive a diagnosis or appropriate treatment, reinforcing the structural challenges identified in our study [10]. In Japan, many migraine patients do not seek medical care [9, 10], a pattern influenced by behavioral and cultural factors such as reliance on over‐the‐counter medications, limited time for medical visits, and prior experiences of being told that “no abnormalities were found” after imaging. These experiences can diminish patients’ motivation to seek care and interact with physician‐side passivity, creating a reinforcing cycle that further reduces treatment opportunities. Internationally, poor adherence and persistence to oral migraine preventive medications have been well documented, with substantial declines in adherence and persistence observed over time [27]. Similar trends are likely in Japan, and the present finding that awareness of conventional preventive medications was high while prescription rates remained low may reflect physicians’ concerns about poor adherence. Limited migraine literacy among patients may contribute to underutilization of effective therapies [26].

Migraine is a major contributor to the global disease burden, as measured by disability‐adjusted life years, particularly among working‐age women [28]. Delayed or inadequate treatment not only reduces patients’ quality of life but also increases societal losses through reduced productivity [29]. Optimizing migraine care is therefore an important issue not only for improving individual patient outcomes but also for addressing broader societal challenges. Recent international advocacy papers have emphasized that improving primary‐care headache management is essential for reducing global disability burden and achieving public‐health goals [30–32]. Given this context, future strategies must extend beyond the simple dissemination of drug information and focus on building a comprehensive educational framework that enables appropriate migraine management across community healthcare. This should include promoting diagnostic and therapeutic algorithms for nonspecialists; sharing evidence on the efficacy and safety of anti‐CGRP monoclonal antibodies and lasmiditan; strengthening collaboration among multidisciplinary professionals such as pharmacists, occupational physicians, and nurses; establishing regional headache care networks; and designing systems that lower the threshold for specialist referral. Continuous educational initiatives led by medical associations may play a particularly important role in improving the quality of migraine care in community settings.

Specialty remained a significant independent predictor of prescribing behavior. Although specialists showed higher triptan prescribing rates in descriptive analyses, specialty was not an independent predictor of triptan prescribing in the multivariable analysis, suggesting that the higher triptan prescribing rates observed among specialists in unadjusted comparisons may be partly attributable to confounding factors—particularly age distribution across specialties—rather than to specialty itself. In contrast, specialists were markedly more likely to prescribe anti‐CGRP monoclonal antibodies (adjusted OR 42.60, 95% CI 14.48–125.30), indicating a strong independent effect on preventive treatment patterns.

This study has several limitations. The response rate was 22.6%, raising the possibility of selection bias. In addition, although respondent characteristics such as age, sex, and specialty were available, no detailed information could be obtained for nonrespondents because the survey was conducted anonymously. Therefore, selection bias cannot be fully excluded. Notably, physicians with a stronger interest in headache care may have been more likely to respond, potentially leading to an overestimation of actual prescribing behavior in community practice. The survey relied on self‐reported data, which may not fully reflect actual prescribing behavior. Moreover, because the study was conducted in an urban area, generalizability to rural regions may be limited. To further assess the potential impact of nonresponse bias, we compared the demographic characteristics of respondents with the publicly available profile of the Kawasaki Medical Association. The age distribution of respondents (mean 58.8 years) was broadly similar to the association’s overall membership, which is known to include a large proportion of mid‐career and senior physicians. The sex distribution (78.1% men) also reflected the male‐dominant composition of the association. In terms of specialty, general internists constitute the largest group within the association, and this pattern was similarly observed among respondents. These similarities suggest that the respondent sample reasonably reflects the broader membership structure, although nonresponse bias cannot be fully excluded. Nevertheless, the detailed analysis of migraine treatment practices by specialty represents a major strength of this study and provides valuable foundational data that can inform future healthcare policy development and targeted educational strategies.

This study demonstrated insufficient awareness and prescription rates of acute and preventive migraine medications among community‐based physicians in Kawasaki, with particularly low levels observed for newer agents such as lasmiditan and anti‐CGRP monoclonal antibodies. The discrepancy between the high awareness and low prescription of certain medications, as well as the low referral rates to specialists, highlights structural challenges that hinder the effective delivery of migraine care within community healthcare settings. Our multivariate analysis further showed that specialty was an independent predictor of anti‐CGRP monoclonal antibody prescribing, whereas specialty was not independently associated with triptan prescribing. Given the substantial societal burden of migraine and the gaps identified in this study, improving access to appropriate treatment requires strengthened education, awareness, and referral‐support initiatives for healthcare providers, including nonheadache specialists, and the establishment of robust collaborative systems with headache specialists.

## Author Contributions

Hisanao Akiyama, Haruka Ono, Yukari Komine, and Yoko Nakano conducted the survey, and Hisanao Akiyama and Yoshihisa Yamano drafted the manuscript.

## Funding

This study was supported by Health, Labour and Welfare Policy Research Grants (Comprehensive Research on Disability Health and Welfare) from the Ministry of Health, Labour and Welfare, Japan (grant number 25GC2003).

## Disclosure

All authors read and approved the final manuscript.

## Ethics Statement

The study was approved by the Institutional Medical Ethics Committee of the St. Marianna University School of Medicine Bioethics Committee (approval no. 6682) and was performed in accordance with the Declaration of Helsinki.

## Consent

Informed consent was obtained from all participating physicians in this study.

## Conflicts of Interest

Hisanao Akiyama received speaker honoraria from Daiichi Sankyo Co., Ltd., Eli Lilly Japan K.K., Otsuka Pharmaceutical Co., Ltd., and Amgen Inc. but no funding related to this manuscript. The remaining authors declare no conflicts of interest.

## Supporting Information

Additional supporting information can be found online in the Supporting Information section.

## Supporting information


**Supporting Information** STROBE Checklist.

## Data Availability

All data are contained in the manuscript.
